# Restoration of vegetation in the Yellow River Basin of Inner Mongolia is limited by geographic factors

**DOI:** 10.1038/s41598-024-65548-6

**Published:** 2024-06-28

**Authors:** Sinan Wang, Xigang Xing, Yingjie Wu, Xuning Guo, Mingyang Li, Xiaoming Ma

**Affiliations:** 1 Yinshanbeilu Grassland Eco-Hydrology National Observation and Research Station, China Institute of Water Resources and Hydropower Research, Beijing, 100038 China; 2 Institute of Water Resources of Pastoral Area Ministry of Water Resources, Hohhot, 010020 China; 3 General Institute of Water Resources and Hydropower Planning and Design, Ministry of Water Resources, Beijing, 100120 China; 4 Water Resources Research Institute of Shandong Province, Jinan, 250014 China; 5 Water Resources Research Institute of Inner Mongolia Autonomous Region, Hohhot, 010052 China

**Keywords:** Vegetation cover, Influencing factor, Geographic detector, Remote sensing, Spatial and temporal variation, Climate sciences, Natural hazards

## Abstract

Studying the relationships between vegetation cover and geography in the Mongolian region of the Yellow River Basin will help to optimize local vegetation recovery strategies and achieve harmonious human relations. Based on MOD13Q1 data, the spatial and temporal variations in fractional vegetation cover (FVC) in the Mongolian Yellow River Basin during 2000–2020 were investigated via trend and correlative analysis. The results are as follows: (1) From 2000 to 2020, the vegetation cover in the Mongolian section of the Yellow River Basin recovered well, the mean increase in the FVC was 0.001/a, the distribution of vegetation showed high coverage in the southeast and low coverage in the northwest, and 31.19% of the total area showed an extremely significant and significant increase in vegetation cover. (2) The explanatory power of each geographic factor significantly differed. Precipitation, soil type, air temperature, land use type and slope were the main driving factors influencing the spatial distribution of the vegetation cover, and for each factor, the explanatory power of its interaction with other factors was greater than that of the single factor. (3) The correlation coefficients between FVC and temperature and precipitation are mainly positive. The mean value of the FVC and its variation trend are characterized by differences in terrain and soil characteristics, population density and land use. Land use conversion can reflect the characteristics of human activities, and positive effects, such as returning farmland to forest and grassland and afforestation of unused land, promote the significant improvement of regional vegetation, while negative effects, such as urban expansion, inhibit the growth of vegetation.

## Introduction

As an essential component of land ecological system, vegetation acts as a “connection” between land, air and water^1–3^. In addition, it is important for the sustainability of this region that the vegetation plays an important role in the prevention of wind and sand fixation, maintenance of soil and water equilibrium, as well as the maintenance of ecology stability^4,5^. Fractional Vegetation Cover (FVC) is a proportion of the land’s vertical projected area relative to the total land area in a statistical region, which is a key index to show the growth of the vegetation on the ground and a fundamental information for describing the ecosystem^6–9^. So, it is very important to dynamically monitor FVC’s time and space variation in drought and semiarid region.

At present, Normal Difference Vegetation Index (NDVI) is employed to estimate FVC based on the merits of being easily constructed and physically significant^10–12^. Many researchers have studied FVC in space and time by means of a binary representation of FVC^13–15^. Kou, et al.^16^ analysed FVC trends, regional contributions, variable FVC stability, and major influential factors including climatic conditions, environmental management, eco-engineering, etc. Shi, et al.^17^ summarized the changes in vegetation cover in semi-arid regions of the globe from 1982 to 2011 and analyzed them with regard to their drivers. Liang, et al.^18^ investigated the spatial and temporal variation of the vegetation cover in the upstream Yellow River by means of tendency and gradient regression, which showed that NDVI was an efficient method for describing the variation of vegetation in Xinjiang County. Jian, et al.^19^ Using Theil-Sen Median and Mann–Kendall Method to analyse the space distribution, monthly and yearly variation of vegetation cover in Yellow River Basin between 1901 and 2100, and then analyse its drivers with bivariate linear regression. The variation tendency of FVC in SSP126, SSP245, SSP370 and SSP585 was investigated. Vegetation cover changes are associated with many factors, including human, topographic, climatic and policy factors^20,21^. It has been found that changes in vegetation cover are strongly associated with differences in urbanization rates, gross domestic product, population density and stages of urban development^22^. For example, changes in land use patterns dominated by human activities profoundly affect vegetation cover change^23^. Many studies on the drivers of FVC change have focused on topographic and climatic factors^24^, with the development of social economy, the influence of human activities on vegetation has become more and more important, and in some areas even exceeds the influence of climatic factors. In addition, the driving force analysis is mostly based on linear correlation analysis, which is unable to quantitatively study the role of each influencing factor. Geoprobe is an analytical model for the detection and quantitative analysis of geographical features and their mutual relations. It has found wide application in geological, medical, agricultural, and biological protection areas^25–28^. Due to the fact that this model is able to fully disclose and quantify the impact of various influential factors on FVC, it has been applied to analyze FVC variation^29–31^.

The Inner Mongolia part of Yellow River Basin is an important part of Inner Mongolia Autonomous Region’s eco-conservation and environment management. Being one of the weakest regions in Inner Mongolia, it has to deal with the problem of water pollution, water scarcity, and water scarcity in addition to the resource-based economy pattern, there are no doubt that it will worsen the conflict of conservation and development^32–34^. So, it is very important to study the time and space change features of FVC in Mongolia area of Yellow River Basin, to understand the current situation, to put forward some countermeasures, to optimize the allocation of land and resources, and to regulate industry structure.

Thus, the research aims of this research are as follows: (1) To explore the features of FVC in Inner Mongolia region from 2000 to 2020 in Inner Mongolia segment of Yellow River Basin; (2) determine the spatial and temporal variation of vegetation due to all kinds of natural and man-made factors. (3) analyses the relationship between geographical factors and vegetation FVC. Then, we can measure the distribution features of FVC and its properties.

## Materials and methods

### Study area

**Figure 1:**
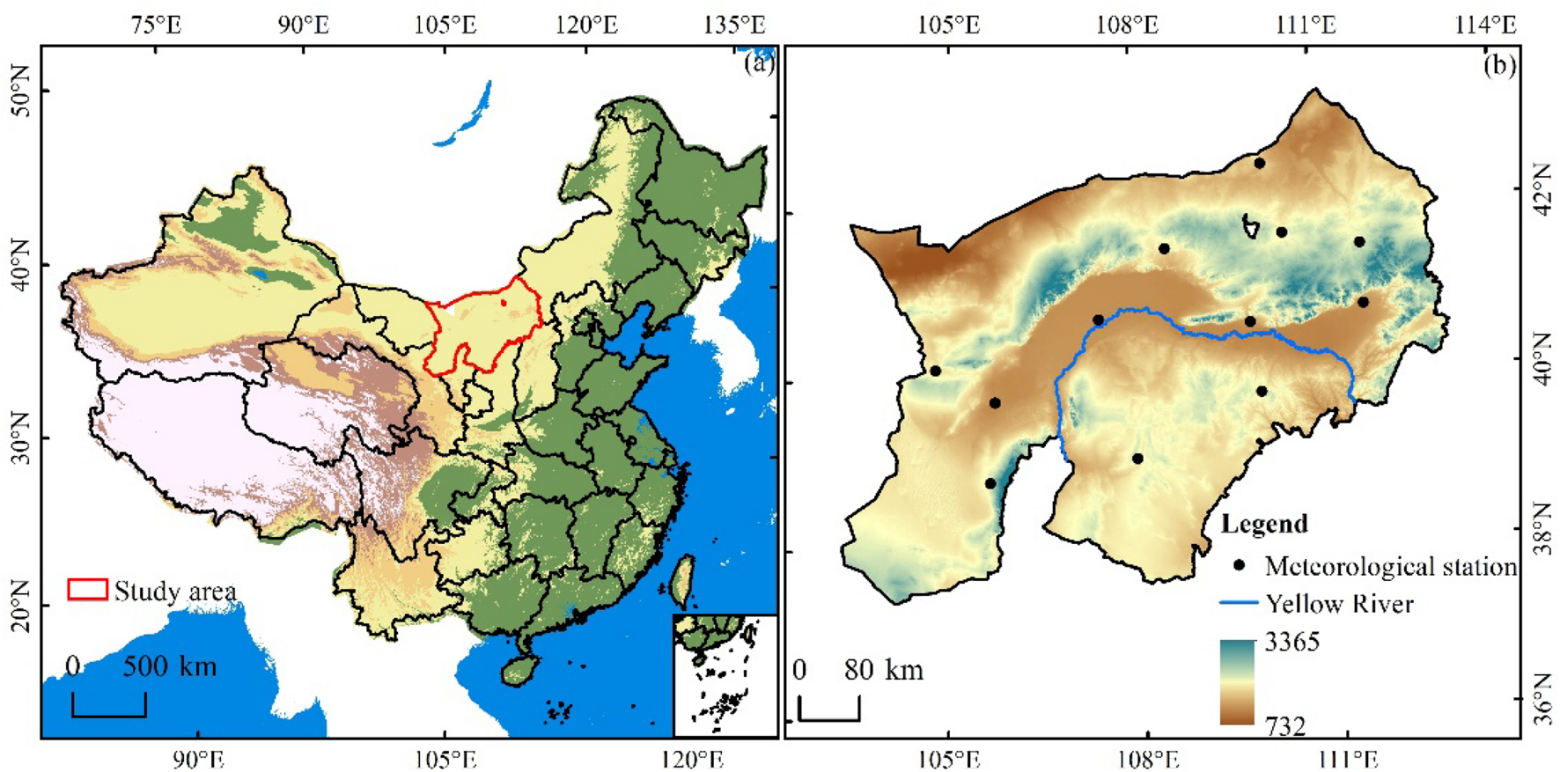
The geographical position of the research region. (**a**) Position of Inner Mongolia Yellow River Valley; (**b**) Numerical Altitude Model.

Inner Mongolia Yellow River Basin lies at the bottom of Yellow River upstream, Great Wall to the north, Tengger and Ulan Buh Deserts, its geographical position is 38° 26′–42′ 50 ′N, 106° 59′–110° 10 ′E. It passes seven towns in Inner Mongolia, covering a total of 830 km and a drainage area of 15.5 × 10^5^ km. The area is dry and semiarid, with an average rainfall of 150–360 mm per year. The eastern part of the country receives more rainfall than the western part. Because of its steep western slope, its altitude varies greatly, resulting in a lot of complicated weather, hydrology and silt (Fig. 1).

### Data sources

The Vegetation Coverage (FVC) is computed using MODIS 16-D NDVI (MOD13Q1) with a space resolution of 250 m. This data set was acquired by NASA (National Aeronautics & Space Administration) (http://lpdaac.usgs.gov), where it has been downloaded for the years 2000–2020 (April–October). Based on this, the MODIS Reprojection Tools were applied to project transform and strip extracting, and lastly, the scope of the research region was cut with the vector edge data in ArcGIS. In addition, the natural breakpoint method was selected after simulation operations, and the slope direction was divided into 10 categories, precipitation, temperature, elevation and slope into 9 categories, soil type was reclassified into 5 categories, land use type was reclassified into 6 categories, and all other elements were reclassified into 5 categories, and the slope direction was reclassified into 10 categories, precipitation, temperature, elevation and slope into 9 categories. Based on ArcGIS 10.2 software to create a fishing net tool, then create a 1 km × 1 km fishing grid, a total of 9865 center points were generated as sampling points to extract the X and Y attribute values corresponding to each index, and finally imported into geodetector for processing.

The weather data are collected from China Meteorological Data Network (www.data.cma.cn/), and the annual mean temperature and rainfall from 12 weather stations in Inner Mongolia Yellow River Basin during 2000–2020 are collected. Using Kriging Technique, we get rid of the lost or abnormal data from each station, and get the same result as NDVI data.

This research chooses every drive’s data into the model, and the data source is presented in Table 1.

**Table 1:** Driving factors of FVC.

Type	Driving factor	Index	Unit	Resolution	Data resource
Natural factor	X1	Mean annual precipitation	°C	1 km	China Meteorological Data Service Center http://www.nmic.cn/
	X2	Mean annual temperature	mm	1 km	
	X3	Elevation	m	30 m	SRTM 30mDEM Data Acquired by NASA http://nasasearch.nasa.gov/
	X4	Slope		30 m	
	X5	Aspect		30 m	
	X6	Drainage density		1 km	National Geographic Information Resources Directory Service System https://www.webmap.cn/
	X7	Soil type	–	1 km	Resource and Environmental Science and Data Center http://www.resdc.cn/
Anthropogenic factor	X8	GDP density		1 km	Inner Mongolia Statistical Yearbook http://tj.nmg.gov.cn/tjyw/jpsj
	X9	Population density	Person/km^2^	1 km	Inner Mongolia Statistical Yearbook http://tj.nmg.gov.cn/tjyw/jpsj
	X10	Traffic density		1 km	National Geographic Information Resources Directory Service System https://www.webmap.cn/
	X11	Land use type		1 km	Data Center for Resources and Environmental Sciences, Chinese Academy of Sciences http://www.resdc.cn/

### Methods

#### The NDVI based dimidiate pixel model

To decrease the effect of non-vegetation zone, we adopt a bivariate model to compute the vegetation cover^35^:

1 $$FVC = \frac{{\left( {NDVI - NDVI_{{{\text{soil}}}} } \right)}}{{\left( {NDVI_{{{\text{veg}}}} - NDVI_{{{\text{soil}}}} } \right)}}$$

Here: FVC is vegetation coverage level, NDVI is VSI, NDVIsoil and NDVIveg are NDVI for non-vegetation and full vegetation. Based on the present condition of the vegetation cover, NDVI was chosen as NDVIsoil and NDVIveg with a cumulative rate of 5% and 95%.

#### Variation trends

In order to quantitatively reflect the spatial and temporal changes of vegetation cover in the study area, the inter-annual trend of FVC was calculated using a one-dimensional linear regression on an image-by-image metric basis, with the following equation^36^:

2 $$\theta_{{{\text{slope}}}} = \frac{{n \times \sum\limits_{i = 1}^{n} {\left( {i \times FVC_{{\text{i}}} } \right) - \sum\limits_{i = 1}^{n} i \times \sum\limits_{i = 1}^{n} {{\text{FVC}}_{i} } } }}{{n \times \sum\limits_{i = 1}^{n} {i^{{2}} - \left( {\sum\limits_{i = 1}^{n} i } \right)^{2} } }}$$

where *FVC*_*i*_ and *FVC*_*j*_ are the vegetation coverage in year *i* and *j*, respectively.

#### Correlation analysis

Correlation analysis can reveal the closeness of the interrelationship between two or more factor variables.

3 $$R = \frac{{\sum\limits_{{i = {0}}}^{n} {\left( {x_{i} - \overline{x} } \right)\left( {y_{i} - \overline{y} } \right)} }}{{\sqrt {\sum\limits_{i = 0}^{n} {\left( {x_{i} - \overline{x} } \right)^{2} } } \sqrt {\sum\limits_{{i = {0}}}^{n} {\left( {y_{i} - \overline{y} } \right)^{2} } } }}$$

where *x*_*i*_* indicates the FVC values in year i*, *y*_*i*_ indicates the Driving factor values in year i, $\overline{x}$, $\overline{y}$ is the average of FVC and driver factors, respectively.

#### Geographic detector

Geographic Probe Model is a kind of statistic approach that can measure the difference in space and discover its motive power. The fundamental theory of this paper is to determine the degree of similarity between two variables in space from the point of view of heterogeneous space level. The model is composed of four types. This thesis focuses on the application of the Element Detector and the Interactive Detector.

1. Factor detection: used to detect the influence of driving factor X on FVC changes, represented by q^37^.
  4 $$q = 1 - \frac{{\sum\limits_{h = 1}^{L} {N_{h} \sigma_{h}^{2} } }}{{N\sigma^{2} }} = 1 - \frac{SSW}{{SST}}$$
  where: *q* is the explanatory power of geographical factor X; L is the classification or partition of the dependent variable FVC or geographical factor X; h is the partition variable (*h* = 1,… ,L); Nh and σ^2^h are the sample size and variance of subregion h, respectively. N and σ^2^ are the number and variance of the whole study area, respectively.

In this case, h = 1,2…, L is the grading or division of the variable Y or Factor X, N and Nh are the number of cells in the entire area and in the entire area, and SSW and SST are the sum of the inner and outer areas, respectively. The value of q is between [0,1] and the greater the value, the more strongly the driver is affected by FVC.

(2) Interaction Detection: Used to determine the interaction among the drivers, that is, if the co-driving forces X1 and X2 have an impact on FVC, or if they affect FVC independently. Interactions have been classified in 5 groups (Table 2).

**Table 2:** Type of interaction between two factors.

Interaction type	q value relationship
Non-linear reduction	q(X_1_ ∩ X_2_) < min(q(X_1_), q(X_2_))
Single factor non-linear reduction	min(q(X_1_), q(X_2_)) < q(X_1_ ∩ X_2_) < max(q(X_1_),q(X_2_))
Bi-factor enhancement	q(X_1_ ∩ X_2_) > max(q(X_1_), q(X_2_))
Independent	q(X_1_ ∩ X_2_) = q(X_1_) + q(X_2_)
Non-linear enhancement	q(X_1_ ∩ X_2_) > q(X_1_) + q(X_2_)

## Results

### Temporal and spatial variation characteristics of FVC

**Figure 2:**
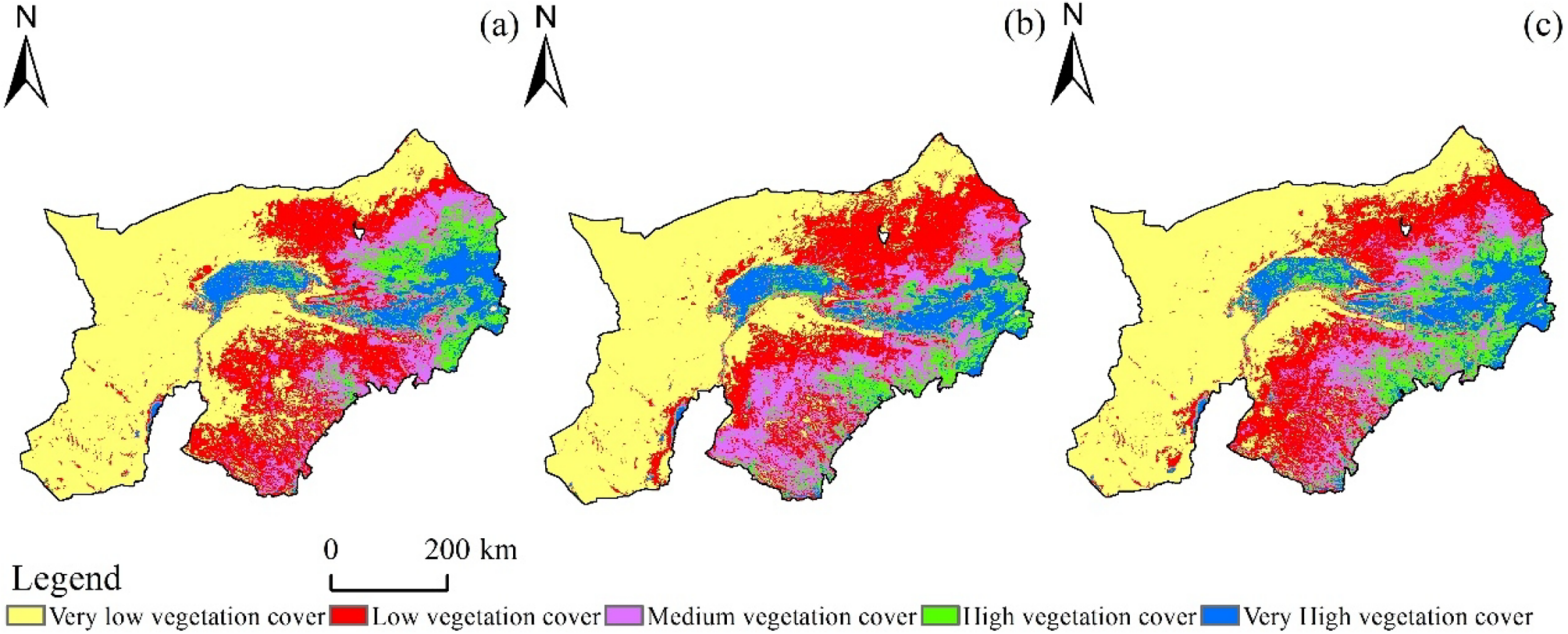
The Spatial Distribution of Vegetation Cover During 2000–2020 in Mongolian Part of Yellow River Basin (**a**) 2000; (**b**) 2010; and (**c**) 2020.

Based on the data of the vegetation cover in 2000–2020, it is found that the vegetation cover is mostly in Yellow River irrigated region and less in west desert (Fig. 2). The vegetation cover in 2000 is the least, and the middle and low-middle cover is comparatively high. The percentage of low-medium vegetation cover in 2010 was higher than that in Ordos City and in Muu Sandy Land. In 2020, FVC will be significantly improved, and the vegetation coverage tends to be stratified from northwest to southeast. In the southeast, including Wuchuan County, Tumt Left Banner and Tumt Right Banner, FVC will increase significantly compared with the previous period.

#### Temporal variation trend of vegetation FVC

**Figure 3:**
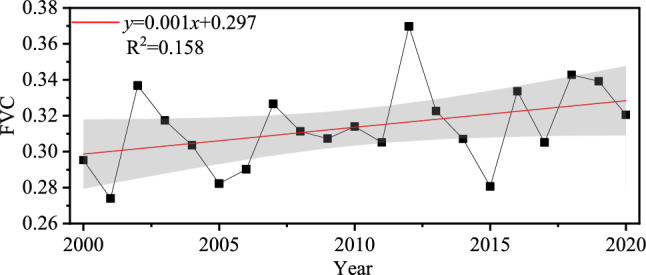
Interannual temporal variation of vegetation FVC.

Based on the yearly mean FVC between 2000 and 2020, we get the yearly variation tendency of the research region (Fig. 3). It is found that the mean increase speed of FVC in the last 21 years is 0.001/a, which is generally increasing (R^2^ = 0 158) with little variation. The mean FVC rose from 0. 286 in 2000 to 0. 312 in 2020. The highest point was 0. 347 in 2012.

#### Spatial variation trend of vegetation FVC

**Figure 4:**
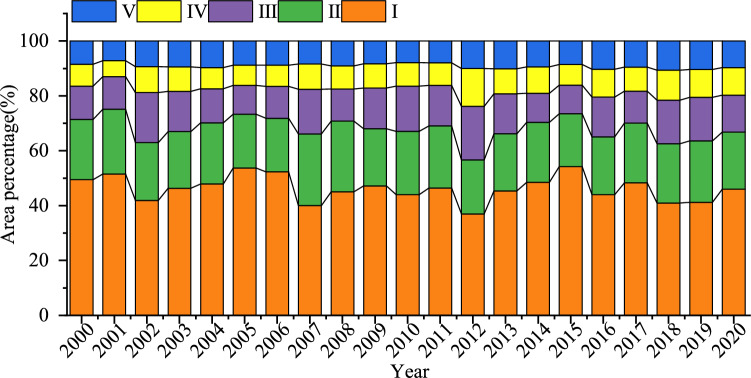
Area proportion of vegetation FVC of different grades.

Figure 4 shows that the overall proportion of vegetation coverage of each grade in the Mongolian section of the Yellow River Basin is successively Very low vegetation cover > low vegetation cover > medium vegetation cover > high vegetation cover > very high vegetation cover. The proportion of very low vegetation cover decreased from 49.45% in 2000 to 45.96% in 2020. The area with low coverage decreased from 21.94% in 2000 to 20.80% in 2020. The average annual area of medium and high vegetation coverage was 13.90% and 8.96%, respectively. High coverage showed a significant increasing trend, from 8.46% in 2000 to 9.66% in 2020.

**Figure 5:**
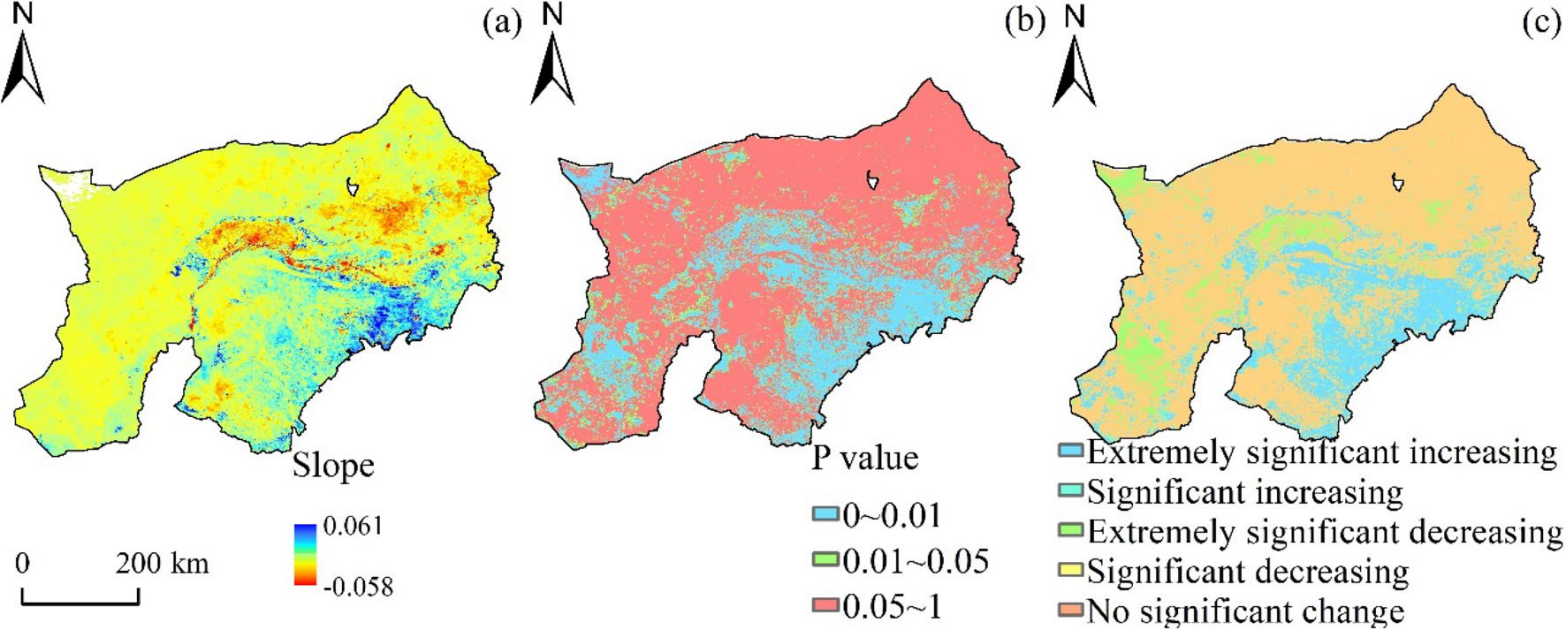
Spatial trend and significance distribution of FVC. (**a**) FVC trend spatial distribution, (**b**) FVC significance spatial distribution, and (**c**) FVC significant trend spatial distribution.

The variation tendency of FVC was from (− 0.058 to 0.061)/a, and it was found that the region had an upward tendency of 56.35% (Fig. 5). The FVC in the last 21 years was significantly different in 30 91% of the region, and 20. 78% of all regions had an obvious rising tendency. The vegetation cover in 39.38 percent areas was significantly reduced. The growth rate of obvious, relative and extreme is over 5%, of which the obvious growth rate is 15.34%, while that of obvious growth is 5.44%. The most important decrease occurred in reducing zones, while the percentage of very important decreases was comparatively small. From the space distribution, the vegetation cover in northwestern part of Yellow River Basin was reduced, and the southeastern part was more obvious.

### Change of vegetation coverage transfer matrix

**Figure 6:**
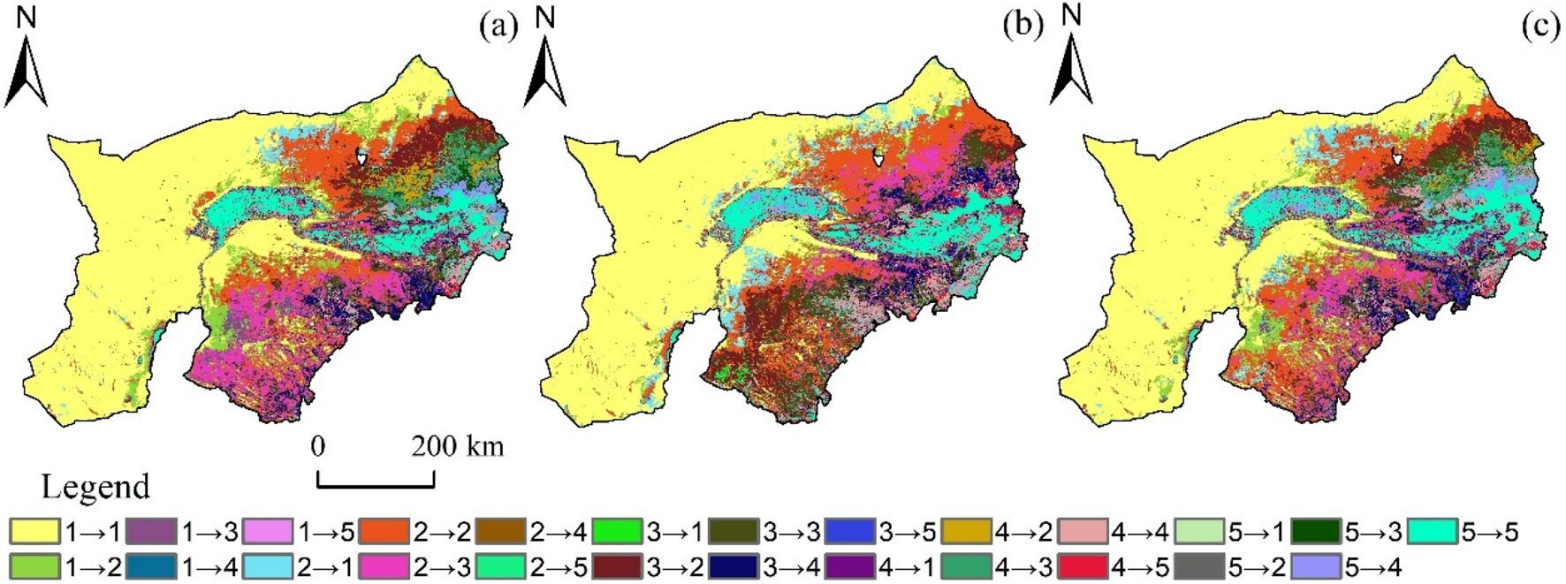
The FVC space transfer matrix varies with time. (**a**) the period from 2000 to 2010; (**b**) from 2010 to 2020; and (**c**) from 2000 to 2020. Note: 1 is Extremely Low Vegetation Cover, 2 is low, 3 is Middle, 4 is High, and 5 is Very High. → for the direction of transfer.

From 2000 to 2010, there were frequent transformations between FVC levels, mainly between very low FVC and low FVC (Fig. 6a). From 2000 to 2010, the proportion of total transferred area of vegetation cover was 34.12%, among which the proportion of low-coverage transferred area was the highest 10.65%, and the main converted to medium coverage was 7.77%. However, 11.81% of the area of other vegetation coverage levels turned to low vegetation coverage, mainly due to very low coverage. The outflow area of very low vegetation coverage was 7.38%, and the outflow area was mainly low vegetation coverage and medium vegetation coverage, accounting for 6.25% and 1.07%, respectively. The main transfer type of the middle FVC is low FVC. The area with very high vegetation cover did not change was 5.84%, and the main transfer type was high vegetation cover (1.57%).

From 2010 to 2020, the proportion of total transferred area of vegetation cover is 26.24%, among which the proportion of low-coverage transferred area is the highest 9.35%, and the main converted to moderate vegetation cover is 4.75%. However, the area of other vegetation coverage levels converted to low vegetation coverage was 7.06%, mainly from the medium vegetation coverage (Fig. 6b). The proportion of the transferred area of the middle FVC was 9.21%, and the main part of the transferred area was low FVC and high FVC, accounting for 4.54% and 3.84% respectively. The proportion of area transferred out by high vegetation coverage was 5.48%, the proportion transferred in was 5.53%, and the main type transferred in was medium vegetation coverage. The conversion area of extremely high vegetation coverage was 3.03%, and the main conversion type was g high vegetation coverage, among which 6.63% did not change.

From 2000 to 2020, the proportion of total transferred area of vegetation cover is 31.54%, among which the proportion of low-coverage transferred area is the highest 10.31%, and the main converted to medium vegetation cover is 5.74%. In contrast, 9.17% of the area with other vegetation coverage levels was low, mainly due to very low vegetation coverage (Fig. 6c). The proportion of the transferred area of the middle FVC was 7.72%, and the main part of the transferred area was low FVC and high FVC, accounting for 3.04% and 3.62% respectively. The proportion of transferred area with high vegetation coverage was 7.12%, and the main transferred type was medium vegetation coverage. The conversion area of extremely high vegetation coverage was 3.36%, and the main conversion type was high vegetation coverage, among which 6.29% did not change.

### Future trends of vegetation coverage

**Figure 7:**
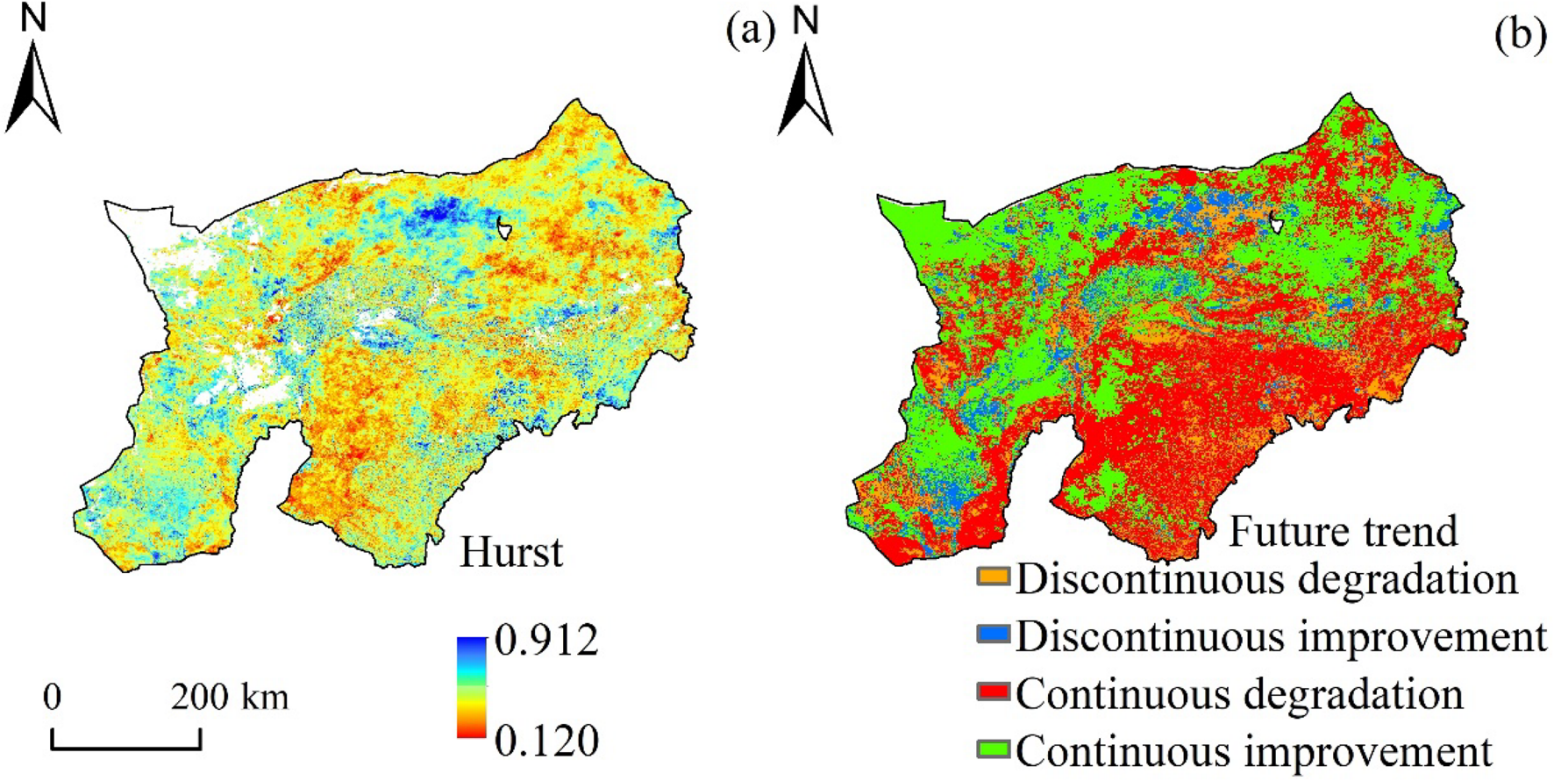
Prediction of Hurst index spatial distribution and future change trend of vegetation coverage. (**a**) Hurst spatial distribution, (**b**) FVC future change trend prediction.

The Hurst index of vegetation coverage in Mongolia section of the Yellow River Basin during 2000–2020 ranged from 0.120 to 0.912 (Fig. 7a). Among them, 23.52% of the regions showed positive persistent changes (H > 0.5), while 76.48% of the regions showed reverse persistent change (H < 0.5). By superimposed analysis of Hurst index and trend change (Fig. 7b), it was found that the area of continuous improvement of vegetation accounted for 37.74% of the total area of the study area, mainly distributed in the north bank of the Mongolian section of the Yellow River basin. At the same time, the area of vegetation change from degradation to improvement accounted for 8.97% of the total area, mainly distributed in part of Hetao irrigation area, the west of Alashan Left Banner and the north of Ulat Zhong Banner. The area of vegetation change from improvement to degradation accounted for 12.88% of the total area, scattered in the middle of the study area. In addition, 40.41% of the vegetation showed a trend of continuous degradation, mainly distributed in the Mu Us Sandy Land in Ordos, Kubuqi Desert and Wuhai City in the west.

### Contribution of geographic factors to vegetation FVC

**Figure 8:**
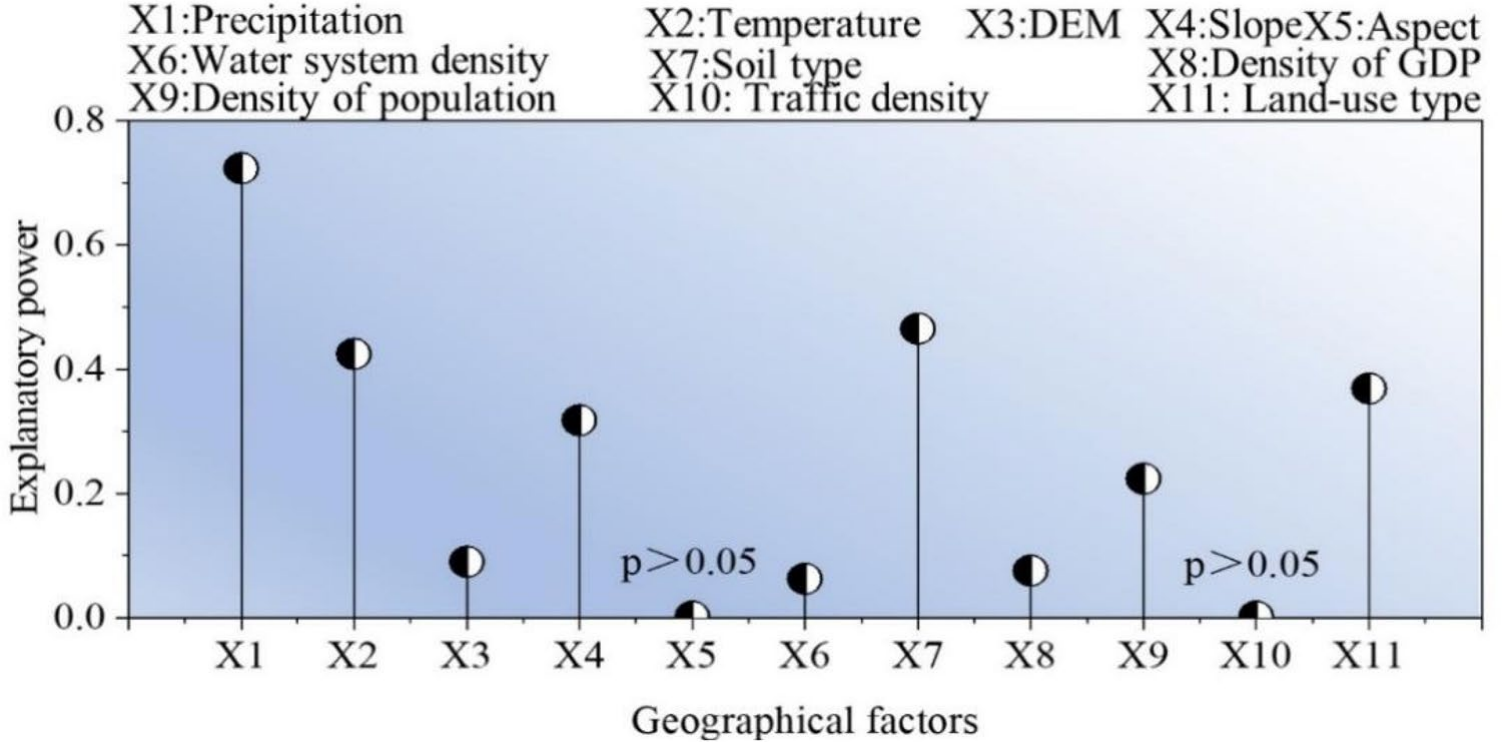
FVC Geographical Survey Results during 2000–2020 in Inner Mongolia Section of Yellow River Basin.

Based on the analysis of climatic, topographic, hydrological, soil and human activities, the author chose 11 geographical elements: rainfall, temperature, altitude, gradient, gradient, water system density, GDP density, population density, traffic density, and land-use patterns. Then, a geoprobe model was applied to analyze the influence of geography on FVC of vegetation (Fig. 8).

The results indicated that the sequence of geographical factors which could account for FVC in Inner Mongolia area of Yellow River Basin were as follows: precipitation (0.723) > soil type (0.465), temperature (0.424) > land-use type (0.369) > gradient (0.318) > population density (0.224) > altitude (0.09) > GDΡ (0.076) > water-system density (0.063) > gradient (0.002) = transportation density (0.002). The *q* values of each factor passed the significance test. The average explanatory power of precipitation is 72.3%, which is much higher than that of other factors, and precipitation is the main driving factor affecting FVC change in the study area. The explanatory power of soil type and temperature were above 40%, which were the secondary driving factors, altitude, GDP, water system density, gradient and transportation density were all less than 10%, and had the least effect on FVC change in the study area.

**Figure 9:**
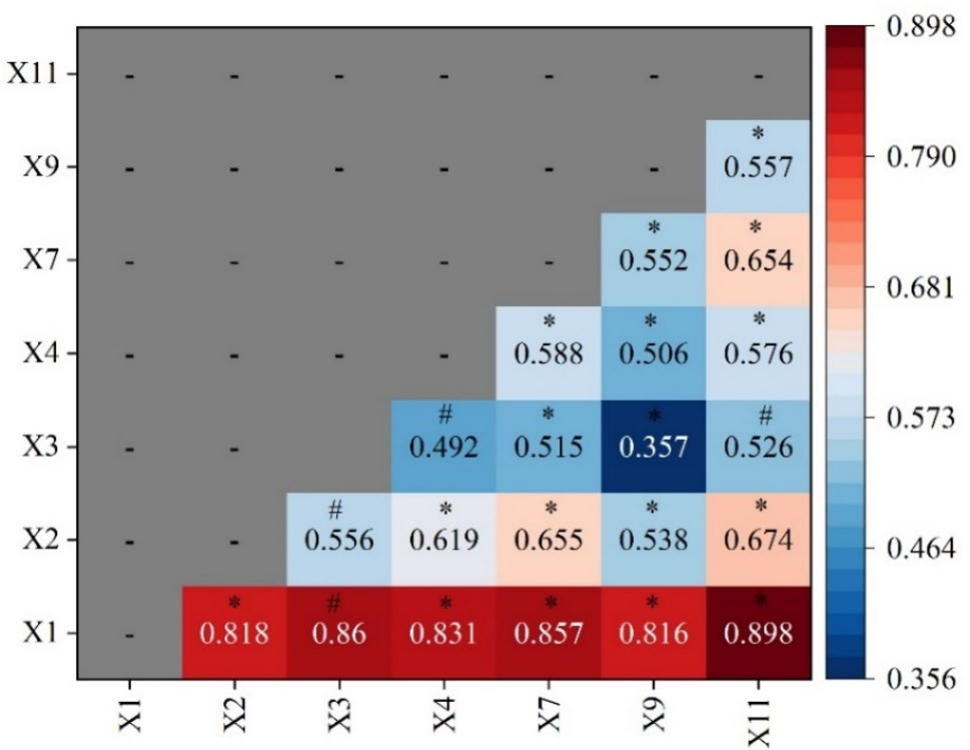
Detection results of FVC geographic factor interactions in the Inner Mongolia section of the Yellow River Basin from 2000 to 2020. Note: The interpretation of X1, X2, X3, X4, X7, X9, X11 is shown in Fig. 8. “*” denotes two-factor enhancement, i.e., q(X1 ∩ X2) > Max(q(X1), q(X2)) q(X1 ∩ X2) > Μax (q(X1), q(X2)); “#” denotes nonlinear enhancement, i.e., q(X1 ∩ X2) > q(X1) + q(X2)q(X1 ∩ X2) > q(X1) + q(X2); “−” denotes no value.

Based on the mutual detecting result, the test results of the main driver and the secondary driver are chosen (Fig. 9), and it can be found that elevation, precipitation, elevation, temperature, slope, elevation and land use type and elevation show nonlinear enhancement; and the interaction results of the rest of the factors show two-factor enhancement. The effect of the effect of rainfall on every element is more significant than that of other factors, which indicates that rainfall is a major controlling factor. The results showed that altitude and population density had an indirect influence on FVC. Overall, while the first and second drivers had greater influence on FVC in Inner Mongolia segment of Yellow River Basin.

### Analysis of geographical factors of vegetation FVC

In order to further analyze the correlation between geographical factors and vegetation FVC in the Yellow River Basin of Inner Mongolia, the above driving factors such as precipitation, temperature, DEM, slop, soil type and Density of population were selected. Correlation analysis and partition statistics were used to explore the distribution characteristics of FVC with the change of each factor attribute.

#### Correlation between vegetation FVC and climate factors

**Figure 10:**
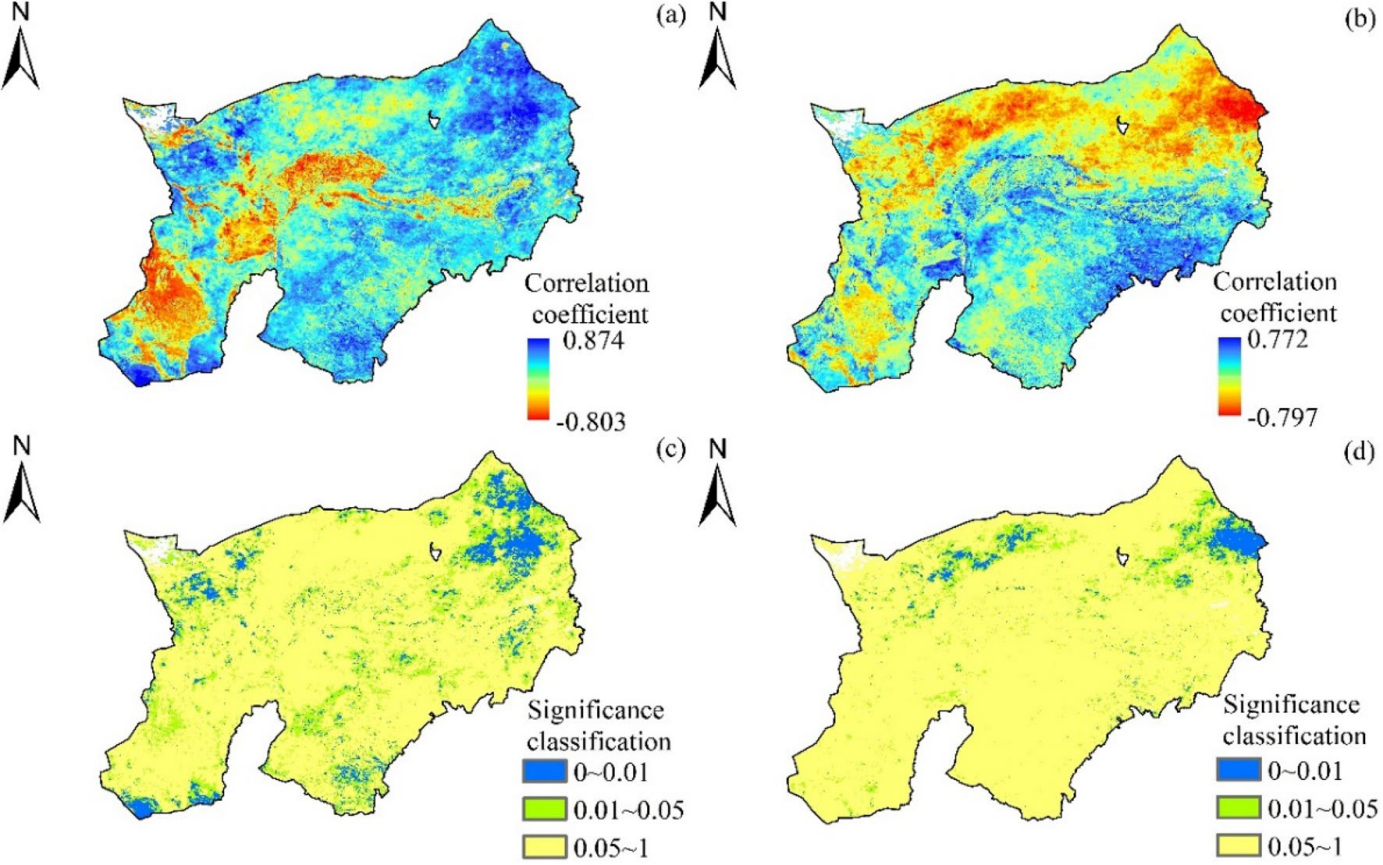
Correlation coefficients and significance of FVC with climate factors. (**a**), (**c**) Correlation and significance of rainfall and FVC, (**b**), (**d**) Correlation and significance of temperature and FVC.

The FVC is positively correlated with rainfall and temperature in Mongolian part of Yellow River Basin (Fig. 10). FVC correlated with rainfall was 0.803–0.847, and the corresponding area was in northwestern and southeastern part of Yellow River Basin. Among them, the positive correlated region was 83.24%, and 28.64% was up to 0.05. It was found in northeastern area. Negative correlated region was 16.76%, while significantly negative correlated region was only 8.45%. The FVC was found to be in a range of 0.78–0.772. The positive and negative correlated regions occupied 60. 34% and 39. 66%. The positive and negative correlated regions occupied 9. 67% and 6. 34%.

#### Topographic factor analysis of vegetation FVC

**Figure 11:**
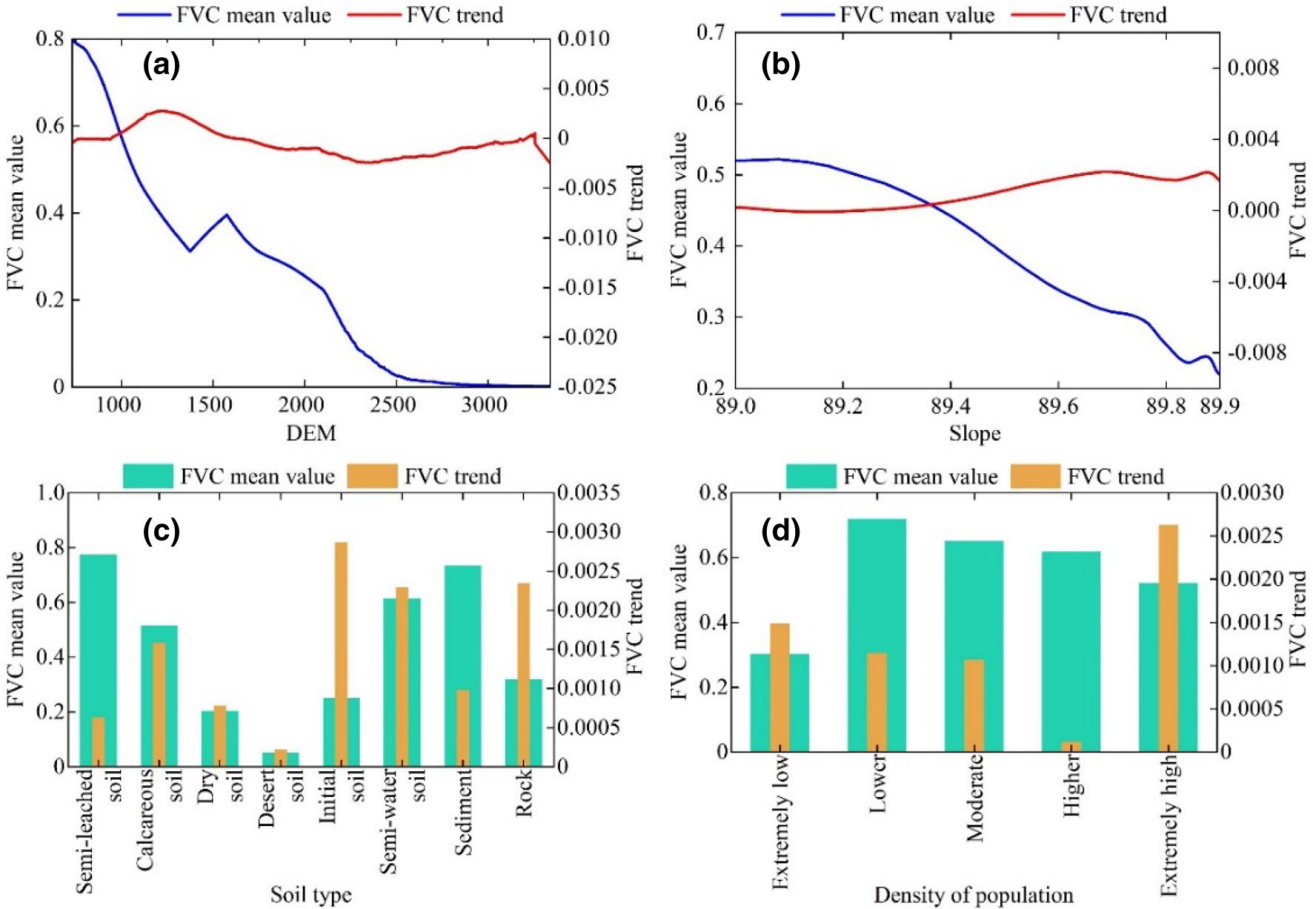
The relationship between FVC mean value and variation trend and factor analysis. (**a**) elevation, (**b**) slope, (**c**) soil type and (**d**) population density.

Using 100 m altitude and 2 degrees gradient as intervals, FVC mean and FVC variation tendency (Fig. 11a), we can see that the mean FVC shows a feature of “fluctuation at first, then slow up, and then decline”, while FVC’s variation tendency is “first fluctuation, then decline”. The variation tendency of FVC is “rising and falling”. The average FVC near the top of the mountain (less than 1000 m) is near the peak, and the growing speed reaches the highest point at 0.0125/a with big fluctuating amplitude. In the mid-elevation region (1000–2000 m), the average FVC rises to 0.396, and then becomes sharply reduced. Both are steady at high altitude (over 2000 m), while FVC falls within the low range.

In terms of slope, the mean value of FVC decreases steadily with the increase of slope (Fig. 11b); the trend of change is characterized by a “sharp decline”. At 89–89.4°, the mean value of FVC is in the high value range, and the deceleration of FVC decreases slowly; at 89.4–89.8°, the degree of deceleration of FVC is larger; when the slope is > 89.8°, the growth rate of the mean value of FVC has a period of recovery.

#### Analysis of soil factors of vegetation FVC

Based on the average and tendency of FVC (Fig. 11c), the average FVC of semi-leached soil was 0.773, next was the sediment and semi-water. The FVC in the original soil is the greatest, which grows at 0.0287/a, and the latter is the most in rocky and semi-water, while the average FVC is the least in the desert, and the growing speed is also low. The average FVC is the least in desert soil, and the growing speed is also low.

#### Analysis of human activity factors in vegetation FVC

As can be seen from the mean value of FVC and the trend of change of population density at all levels (Fig. 11d), the mean value of FVC corresponding to the areas with population density Lower, Moderate, and Higher is larger, in which the highest value of Lower is 0.719; the mean value of FVC of the areas with population density Extremely low is at a lower level, but the trend of change of FVC is larger, in which the Extremely low has a growth rate of 0.0014/a.

#### Analysis of land use change of vegetation FVC

From the Yellow River Basin Inner Mongolia section of the land use type changes can be seen (Table 3), 2000–2020 water bodies, construction land area increased significantly, while arable land, forest land, grassland, unutilized land is significantly reduced, the transfer of land use is mainly dominated by the transfer of arable land, grassland. Among them, the area of arable land transferred to grassland is the largest, which is 10329 km^2^; the main transfer mode of forest is grassland and arable land transferred to forest, and its transfer area is 3278 km^2^ and 704 km^2^ respectively; the main transfer mode of unutilized land is grassland, and the transfer area is 14733 km^2^. In a word, the area of returning arable land to forests and grasslands reaches 6334 km^2^, and the area of unutilized greening reaches 16887 km^2^, and the area of urban expansion is 16887 km^2^, and the area of urban expansion is 16887 km^2^. In total, the area of returning farmland to forest and grassland amounted to 6334 km^2^, the area of greening unutilized land amounted to 16887 km^2^ and the area of urban expansion was 5738 km^2^.

**Table 3:** Transformation Matrix of Land-Utilization Pattern in Mongolian Yellow River Basin during 2000–2020.

	2020						
	Farmland	Forest	Grassland	Water	Urban land	Unused land	Total
2000							
Farmland	*	704	5630	620	2121	1254	10,329
Forest	932	*	2634	141	236	407	4350
Grassland	7771	3278	*	1763	2495	14,171	29,478
Water	594	119	1758	*	165	843	3479
Urban land	1899	162	1486	99	*	277	3923
Unused land	1464	690	14,733	923	721	*	18,531
Total	12,660	4953	26,241	3546	5738	16,952	70,090

“*”Indicates no change in land use type.

## Discussion

### Characterization of the spatial and temporal evolution of FVC

From 2000 to 2020, FVC in Inner Mongolia part of Yellow River Basin had a general upward tendency, which was in agreement with the results of Wang et al.^38^ and Wang et al.^39^. On the whole, the vegetation situation in most areas of the Yellow River basin in Inner Mongolia is getting better and better. Among them, the improvement area accounts for 56.35% of the whole study area, while the degradation area only accounts for 43.65%. The improvement area of FVC was mainly distributed in the middle of the study area. However, the extent of degradation is mainly in the eastern urban areas. To be specific, Hetao Plain’s agriculture irrigation region lies in the warm dry belt. Because of the beneficial effects of the Yellow River’s irrigation and human-made agriculture, it has become a cultivation ground for wheat, rice, and other crops, with a wide range of vegetation coverage compared with those in the nearby deserts. The large-scale construction of sandy soil and Three-North Protective Project in Kubuqi Desert and Maowusu Desert have made significant improvements in vegetation coverage. This leads to a shift of the centre of mass of vegetation towards the south in agriculture and in the desert regions^40^. Among them, Damao Banner and Erdos are important regions for eco-engineering such as reconversion of cropland into woodland and grass and forestation. Zhao et al.^41^ shows that Maowusu Sandland is the best place to recover vegetation, which confirms the trend of the centre of mass of vegetation in Inner Mongolia Yellow River Basin. The Daqingshan, Wulasan and Wuyushan Hills have a high altitude and a sharp topography, which is rich in black calcareous soil, which gives rise to highly depressed forests^42^. Due to the high density of people in Baotou and other provinces, lots of farmland and grass have been taken up by the development of the city, which has resulted in the decrease of the vegetation^43^.

### Driving factors affecting FVC changes

The results show that there is a significant positive correlation between precipitation and temperature in the northwestern part of Yellow River Basin of Inner Mongolia, where there is a lack of rain, and the water demand for big herbs is mostly from rainwater, so it is more sensitive to rainfall^44^. The southeast Inner Mongolia portion of Yellow River Basin is not subject to water constraints as it has abundant rainfall, so it has a higher dependence on temperature^45^. The vegetation in Inner Mongolia part of Yellow River Basin has distinctive landform, soil, population density, and land utilization, which is dominated by forestry and semi-leached soil in mid-and high-altitude mountainous areas, with little human activity^46^. There is a great deal of building, farmland and desert land in middle-and low-altitude plain, which leads to the different status and speed of vegetation cover in mountain and valley^47^. The characteristics of human activities can be reflected by land use changes, as of 2020, the area converted to forest and grassland is 4953 km^2^, 26241 km^2^, respectively, and the average growth rate of FVC is 0.001/a, which indicates that the implementation of policies such as returning farmland to forest and grassland, afforestation, and desert management effectively promotes the increase of vegetation cover in the Inner Mongolia section of the Yellow River Basin^48–50^.

All in all, the vegetation cover of Inner Mongolia Yellow River Basin is influenced by various geographical factors, and there is mutual interaction among them^51^. Topography affects vegetation distribution mainly by changing the water and heat conditions in the environment. As an important topographic factor, elevation has an important impact on the growth process of surface vegetation, and is the main factor affecting the distribution of water and heat conditions in mountainous areas, which has a certain complexity. With the increase of altitude, the temperature drops, solar radiation and wind speed increase, precipitation and relative humidity in local areas increase first and then decrease, and soil types show significant differences, forming the change of environmental gradient, resulting in different plant types and growth characteristics at different altitudes^52^. Land use type also plays a crucial role in the growth of vegetation in the study area, accounting for 36.9% of vegetation change. Among them, the desert region has poor climatic conditions and less precipitation, which inhibits the growth of vegetation to a large extent. Different soil types have different topographic characteristics, soil and land use patterns, which can significantly affect the distribution of vegetation. Soil type can explain 46.5% of the vegetation change in the study area, and has a significant effect on the vegetation change in the study area. The high vegetation coverage area is mainly distributed in the plain area around the main stream of the Yellow River, with gentle slope, adequate hydrothermal conditions and relatively suitable altitude, which is conducive to vegetation growth. In this way, the variation of the earth’s natural cover may lead to a change in the characteristics of the soil, for example, by restoring the slope to the woodland, and the grass can increase the amount of precipitation and the ability to retain moisture, thus reducing the loss of the soil, thereby efficiently transforming it into vegetable water, thereby encouraging the growth of the plant^53–55^. Large scale vegetation recovery results in the variation of terrestrial and atmospheric interaction, which is beneficial for the recovery of vegetation^56^. In such a process of accumulation, man’s action can affect the orientation of the vegetation.

### Uncertainties and limitations

In this research, we have investigated the variation of vegetation cover in Inner Mongolia area of Yellow River Basin with no consideration given to the influence of moisture, sunlight and water content. Moreover, it is difficult to quantify human features by means of population density and land-use change. Thus, it is possible to study more closely the impact of human behaviors on vegetation by integrating many kinds of natural elements in order to study the variation of vegetation in a long-term sequence.

## Conclusions

Using MOD13Q1 data, we investigate the time and space variation of FVC in Inner Mongolia area during the past 21 years. The conclusions are as follows:

1. Between 2000 and 2020, the recovery of plant cover in Mongolia part of Yellow River Basin was good, FVC increased by 0.001/a, and it was highly distributed in southeastern part of Mongolia. The ratio of extreme significance and obvious growth rate is 31.19 percent, which shows that the conversion of agricultural land into forestry and nature forest conservation in Mongolia part of Yellow River Basin has obvious influence on the recovery of native plants.
2. Geographic explanation ability of FVC is significantly different from that of Mongolia Yellow River Basin. Rainfall, soil type, air temperature, land-use pattern and gradient are the major drivers of FVC’s space distribution, and its explanation ability is above 0.2.
3. FVC was correlated with geographic factors in Mongolia Yellow River Basin. The correlative factors of rainfall, temperature and FVC were positively correlated. The FVC average and the change tendency were influenced by the topography, the soil, the density and the utilization of the land. The change of land use can reflect the features of mankind, and it can improve the local vegetation obviously.

## Data Availability

The datasets used and/or analysed during the current study available from the corresponding author on reasonable request.
